# Feasibility of a Novel Sparse Orthogonal Collimator–Based Preclinical Total Marrow Irradiation for Enhanced Dosimetric Conformality

**DOI:** 10.3389/fonc.2022.941814

**Published:** 2022-07-18

**Authors:** Amr M. H. Abdelhamid, Lu Jiang, Darren Zuro, An Liu, Srideshikan Sargur Madabushi, Hemendra Ghimire, Jeffrey Y. C. Wong, Simonetta Saldi, Christian Fulcheri, Claudio Zucchetti, Antonio Pierini, Ke Sheng, Cynthia Aristei, Susanta K. Hui

**Affiliations:** ^1^ Department of Radiation Oncology, City of Hope Medical Center, Duarte, CA, United States; ^2^ Radiation Oncology Section, Department of Medicine and Surgery, Perugia University and General Hospital, Perugia, Italy; ^3^ Department of Clinical Oncology and Nuclear Medicine, Faculty of Medicine, Ain Shams University, Cairo, Egypt; ^4^ Department of Radiation Oncology, University of California Los Angeles, Los Angeles, CA, United States; ^5^ Department of Radiation Oncology, University of Oklahoma, Norman, OK, United States

**Keywords:** TBI, TMI, SOC, HCT, RAO, CBCT (cone beam computed tomography)

## Abstract

Total marrow irradiation (TMI) has significantly improved radiation conditioning for hematopoietic cell transplantation in hematologic diseases by reducing conditioning-induced toxicities and improving survival outcomes in relapsed/refractory patients. Recently, preclinical three-dimensional image–guided TMI has been developed to enhance mechanistic understanding of the role of TMI and to support the development of experimental therapeutics. However, a dosimetric comparison between preclinical and clinical TMI reveals that the preclinical TMI treatment lacks the ability to reduce the dose to some of the vital organs that are very close to the skeletal system and thus limits the ability to evaluate radiobiological relevance. To overcome this limit, we introduce a novel Sparse Orthogonal Collimator (SOC)–based TMI and evaluate its ability to enhance dosimetric conformality. The SOC-TMI–based dose modulation technique significantly improves TMI treatment planning by reducing radiation exposures to critical organs that are close to the skeletal system that leads to reducing the gap between clinical and preclinical TMI.

## Introduction

Radiotherapy is an important component of bone marrow transplantation condition regimens for hematological diseases (1). For more than 50 years, total body irradiation (TBI) has been a standard of care as a conditioning regimen for the host immune suppression and for the reduction of disease burden to allow donor engraftment (2, 3). Several randomized trials have demonstrated superior outcomes using TBI compared to non-TBI–containing regimens (4, 5). Although TBI-based dose escalation reduces relapse in high-risk patients with leukemia, no benefit in overall survival was observed due to organ toxicity (6). This further emphasized the unmet clinical need for advanced technology.

Hui et al., for the first in the field, have developed a more targeted conformal form of TBI delivery [total marrow irradiation (TMI)] (7). TMI approach is to spare the organs at risk (OARs) and the remaining healthy tissues in the body, maintaining the coverage of target hematopoietic or lymphoid tissues with respect to the standard TBI. The feasibility and early clinical data of TMI were reported both by helical tomotherapy (HT)–based approaches (7–10), conventional Linac using intensity-modulated radiation therapy (IMRT) (11, 12), and volumetric modulated arc therapy (VMAT) using rapid arc approach (Varian Medical Systems, Palo Alto, CA) (13, 14).

Although new clinical technology supported clinical advancement, the relapse rate remains high. For a mechanistic understanding of the role of TMI on engraftment, antileukemic effect, etc., and to envelope future experimental therapeutics, a preclinical mouse model is essential. A film-based two-dimensional (2D) image guidance method identifying organ position and copper compensator was used to develop the first-generation preclinical TMI (15). However, it lacks three-dimensional (3D) imaging to detect targets and organs, generating organ dosimetry such as dose-volume histograms (DVHs), the inclusion of tissue heterogeneity, and the ability to vary dose exposures. Therefore, we developed the second-generation CT image–guided 3D-TMI (16), which, however, showed limited ability to reduce dose to organs that are close to the skeletal system (e.g., lungs and kidney). In this study, we introduce a new concept Sparse Orthogonal Collimator (SOC)–based TMI (SOC-TMI). Comparative analysis of SOC-TMI planning and dosimetry from recently reported 3D-TMI and several clinical TMI plans shows substantial improvements in dosimetric control while accompanying SOC in preclinical TMI platforms.

## Material and Methods

### Clinical TMI Studies

We reviewed published articles, in which patients with leukemia were treated with TMI techniques using either HT (Tomotherapy, Madison, WI) or Linac-based volumetric arc therapy. From the available literature, we selected four papers that were conducted to evaluate whether TMI obtained optimal dosimetric coverage of the PTV and sparing of various organs such as the heart, gut, lungs, kidneys, and liver (8, 9, 13, 14). Furthermore, data from two unpublished clinical TMI studies from the Radiation Oncology Department of the Perugia University and City of Hope Radiation Oncology Center were analyzed. Because target prescription dose varies across centers, we calculated the percentage dose exposure to organs with respect to the prescribed dose. This relative dose exposure is then compared with the relative dose exposure obtained from 3D-TMI and SOC-TMI preclinical models.

### TMI Preclinical Models

The preclinical TMI treatments were performed using the x-ray irradiator (Precision X-Ray, North Branford, CT) in compliance with the current guidelines (17). It has a maximum tube potential of 225 kV. Photons were filtered through a beryllium window with an additional 2.0-mm aluminum filter for imaging and 0.32-mm copper filter for treatment (18). We have used five-mice cases for both TMI preclinical models. The animal was placed in a custom-designed animal holder under isoflurane anesthesia to ensure its immobilization and reproducible positioning. Cone Beam Computed Tomography (CBCT) scans of reference animals in the prone position were acquired using 40-kVp and 3-mA beam settings with a 0.2-mm voxel size. Using velocity, soft tissue organs were identified and contoured for use in treatment planning. Moreover, we contoured the entire body minus the skeletal bones and the spleen to calculate the integral dose to the body. After contouring, images were exported to the planning systems to generate TMI plans.

#### 3D-TMI Preclinical

**Figure 1:**
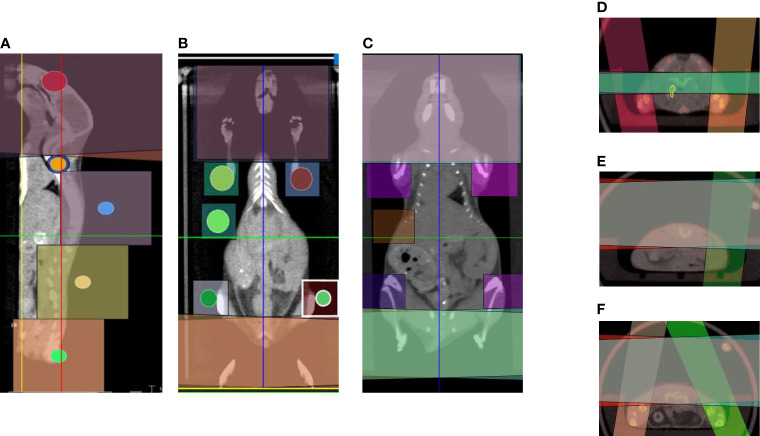
Common beam arrangements of 3D-TMI treatment. Beam arrangement for parallel opposed beams in **(A)** sagittal view. **(B, C)** Coronal views demonstrating different body levels. **(D–F)** Axial view in the thoracic, abdominal, and pelvic level showing beams overlapping.

The 3D-TMI preclinical model was developed as a second-generation TMI preclinical model (16). Mice 3D-TMI preclinical radiation treatment plans are generated by following steps: The whole-body mouse CBCT scan was performed. Next, whole-body CT scans separated into seven regions: head, cervical spine, dorsal spine and spleen, lumber spine, femurs, tibias, and shoulders. Visualization of a projected radiation beam on a 3D CT image allowed for adjustment of beam size and isocenter to cover the target and reduce exposure to adjacent critical organs. Each region has its beam size, isocenter location, and normalization point, and parallel-opposed beams with varied beam size were used to create a homogenized dose. Field matching is achieved by imposing different beam sizes [40 mm × 40 mm (standard), 20 mm × 20 mm (standard), 10 mm × 10 mm (standard), 10 mm × 10 mm (cylinder), and 5 mm × 5 mm (cylinder)], both parallelism and coincidence between the side planes of adjacent fields **(** **Figures 1A–C** **)**. However, the spleen, femurs, and tibias beams are matched with dorsal and lumber beams. Matched beams in those regions have some hot spots because of the intersection between lateral parallelized beams and perpendicular beams **(** **Figures 1D–F** **)**. Mice TMI is a 3D treatment planning with kilovoltage (kV) radiation beams on whole-body CBCT image, and dose distribution and absorbed dose are significantly affected by HU CBCT pixel values, as bone areas absorbed more than 250% of the prescribed dose due to kV-radiation beams (19, 20) (**Figures 3A, B** **)**. The CT-guided Monte Carlo dose calculations accounted for tissue heterogeneity, enhancing accuracy of organ dose evaluation. Detail validation of Monte Carlo–based treatment planning system (TPS) including calculation of dose to medium was previously published (17, 18).

GAFChromic EBT3 film- and dosimeter-based dosimetry was used for the dosimetric validation. Briefly, the film was calibrated for treatment settings at the isocenter up to 5 Gy. After calibration, the film was placed under the mouse, and a tissue prescription dose of 2 Gy was delivered. Afterward, different regions of interest were outlined in the film identifying the exit dose through the spine, lungs, and gut to accommodate density variation in the path of the x-ray. The mean dose measurement was compared with the TPS at the film location to establish an agreement. We used 2 Gy for film validation so that the dose response was in the linear region of the EBT3 film. The overall time of 3D-TMI preclinical planning is approximately 75 min: 15 min for beam placement; 45 min for planning, optimization, and dose calculation; and 25 min for the estimated delivery time.

#### SOC-TMI Preclinical

**Figure 2:**
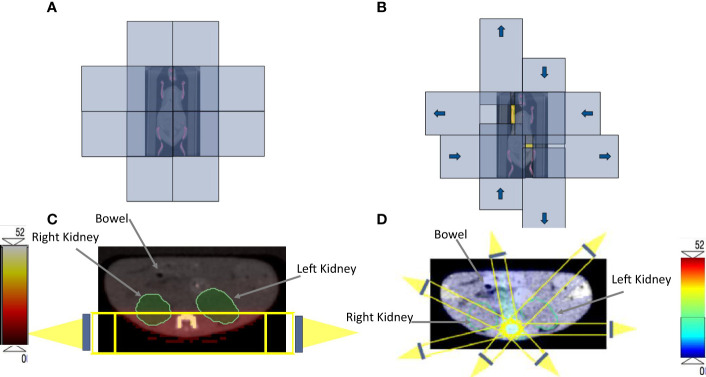
**(A)** The four orthogonal, double-focused tungsten leaf pairs before the optimization. **(B)** The four orthogonal, double-focused tungsten leaf pairs after applying the rectangular components to cover the target volume; the yellow color represents the area that beams can go through to the PTV. Identical axial CBCT image at the abdomen level that is showing the kidneys and bowel for both **(C)** 3D-TMI preclinical model beam arrangements. **(D)** SOC-TMI schematic beams arrangement according to the X-RAD SmART small animal image-guided irradiation system.

Mouse position, immobilization, and CBCT imaging are the same as in the 3D-TMI preclinical model. The SOC system is designed and fabricated with four orthogonal, double-focused tungsten leaf pairs, which is programmed and controlled by the Rectangular Aperture Optimization (RAO) algorithm (21, 22), which solves an inverse optimization problem for IMRT planning. The SOC can be installed on the small animal irradiator (Precision X-Ray, North Branford, CT) with a 3D printed adapter. The plan is uploaded to the SOC control module, which drives tungsten leaf pairs to form rectangular apertures and delivers the dose to mouse bones and spleen while sparing adjacent organs at risk. The SOC plans are utilizing seven equally distributed coplanar fields. In each coplanar field, there are several rectangular components made by tungsten collimators to deliver the dose. **Figure 2A** shows the four orthogonal, double-focused tungsten leaf pairs closed before planning optimization. **Figure 2B** shows how tungsten collimators move to form rectangular components at the coronal angle, and color yellow means the area that beams can go through to the PTV. Overall, each IMRT SOC-TMI preclinical plan uses 61 to 93 rectangles per field to intensity modulate the x-ray fluence. The number of rectangles depends on the size and complexity of the target as a result of RAO inverse optimization. **Figures 2C, D** show SOC-TMI and 3D-TMI schematic beam arrangement according to the X-RAD SmART small animal image-guided irradiation system, respectively. SOC-TMI preclinical model uses convolution/superposition code with a 225-kV x-ray poly-energetic kernel in a distributed multiple GPU framework, as described (21–23) for the beamlet dose calculation; its accuracy in profile dose is below 2% on average from Monte Carlo simulation, but it is faster and more flexible to meet performance requirements for most users. A fast-iterative shrinkage-thresholding algorithm is used to optimize the treatment plan. The beam commissioning data were acquired on the small animal irradiator (Precision X-Ray, North Branford, CT) (21). The beamlet resolution at the isocenter was 1 mm × 1 mm. The dose array resolution was 0.25 mm × 0.25 mm × 0.25 mm. The source-to-isocenter distance (SID) was 30.54 cm. For SOC-TMI plans, the field size is extended to 120 mm × 120 mm. The dose calculation and optimization were performed on a Xeon 40‐core CPU server operating at 3.10-GHz clock with MATLAB. The overall time of SOC planning is approximately 45 min: 25 min for planning, optimization, and dose calculation and 20 min for the estimated delivery time.

## Results

**Figure 3:**
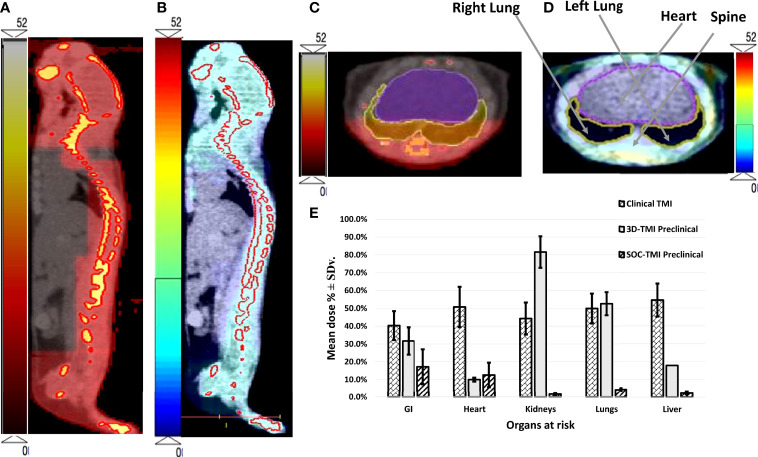
The dose distribution of both **(A)** 3D-TMI preclinical model and **(B)** SOC-TMI preclinical model. **(C, D)** Identical axial CBCT image obtained at the level of the lungs of mouse shows conformal dose distribution for spine, ribs, and sternum for both SOC-preclinical and 3D-preclinical model, respectively. **(E)** Bar chart shown for critical organs—GI, heart, kidneys, lungs, and liver. Three bars in each group represent the mean dose for average of six clinical TMI studies, 3D-TMI preclinical, and SOC-TMI preclinical.

For each of the five mice, median dose percent, average, and standard deviation for organs at risk in both 3D-TMI preclinical model and SOC-TMI preclinical model are listed in **Table 1**. The median dose of TMI clinical and preclinical studies with treatment protocol, treatment technique, and prescription dose and a few fractionation schemes with number of enrolled patients are listed in **Table 2**. A total of eight studies (six clinical TMI studies, average of five mice 3D-preclinical TMI plans, and average of five mice SOC-preclinical plans) were enrolled in this dosimetric analysis, aiming to compare the clinical and preclinical models and to highlight the advantages of the SOC-preclinical model. For the preclinical models, the bar chart of median dose with the standard variation for organs at risk for the average of five mice in both 3D-TMI and SOC-TMI is presented **Figure 3E**. The median dose difference is represented as a percentage of the prescription dose. OARs are the heart, kidneys, liver, lungs, and gastrointestinal (GI). For the six clinical TMI studies, the average median doses of the OARs were approximately 30%–65% of the prescribed PTV dose. Otherwise, the 3D-TMI preclinical model reduced heart, liver, and GI doses compared to clinical studies. Whereas the lungs and kidneys doses were very high due to their proximity to the spine, the median dose was about 52.6% and 81.6% of the prescribed dose, respectively. SOC-TMI preclinical model has more organ dose sparing capability, especially the kidneys and lungs. Dose to the lungs was reduced by 95.8% ± 0.8%, to the kidneys by 98.4 ± 0.5%, and to the liver by 97.7± 0.7% of the prescription dose. GI and heart doses have been reduced by 82.8± 9.8% and 87.4 ± 11.3% of the prescription dose, respectively.

**Table 1:** The median dose with standard deviation of the organs at risks for the 3D-TMI preclinical model versus the SOC-TMI preclinical model.

OARs’ median dose percent in both 3D-TMI and SOC-TMI															
OARs	Bowel %		Heart %						Kidneys %		Lungs %		Liver %	
Plan	SOC	3D	SOC			3D			SOC	3D	SOC	3D	SOC	3D
**m1**	28.3	37.5	6.5			6.511.1			0.9	91.3	4.8	58.3	2.5	22.5
**m2**	27.4	38.0	5			9.3			1.4	70.0	4.6	43.8	1.6	13.8
**m3**	8.6	24.2	20.3			11.3			2.3	89.0	4.7	48.3	1.4	9.8
**m4**	11.9	22.5	18.7			9.2			1.6	76.3	4.1	53.8	3.1	18.8
**m5**	9.7	36.3	12.4			9.1			1.6	81.3	2.9	58.8	2.8	23.8
**Average**	17.2	31.7	12.6			10			1.6	81.6	4.2	52.6	2.3	17.7
**SD**	9.8	7.7	6.9			1.1			0.5	8.8	0.8	6.5	0.7	5.9

**Table 2:** Dosimetric results of median dose in percent of the dose prescription of organs at risks for different clinical and preclinical TMI models.

Study	Clinical 1	Clinical 2	Clinical 3	Clinical 4 (A)	Clinical 4 (B)	Clinical 5	Clinical 6	3D-Preclinical	SOC-Preclinical
**Treatment technique**	IMRT-Linac	HT	VMAT-Linac	HT	VMAT-Linac	HT	HT	3D-Preclinical TMI	SOC-Preclinical TMI
**Prescription dose (Gy)**	12	6	12	12	12	13.5	20	12	12
**Number of planned patients**	3	1	6	4	4	12	8	5	5
**OAR metric**	**Median Dose (%)**								
**Heart**	52	70	46	53	48	35.5	31	10	12.6
**Kidneys**	47	40	45	60	40	33.7	29.7	81.6	1.6
**Liver**	50	70	49	60	54	44	NA	17.7	2.3
**Lungs**	36	57	60	48	50	48.5	32.7	52.6	4.2
**Bowel**	29	NA	49	40	47	36.4	38.9	31.7	17.2

SOC-TMI average integral dose was 53.1% of the prescribed dose, whereas 3D-TMI dose was 70.7% of the prescribed dose. SOC-TMI has shown a significant median dose reduction to the lungs by 48.4% (p = 0.014) and to the kidneys by 80.6% (p = 0.013), but non-significant reductions were observed in the liver and GI by 15.4% and 14.5%, respectively. The heart received a slightly greater median dose by 2.6%.

The dose coverage to the whole-body is shown in a color wash presentation in **Figures 3A, B** for 3D-preclinical TMI and SOC-preclinical TMI, respectively. The color map is the dose level between 0 Gy as minimum to 52 Gy as the maximum dose range in preclinical TMI models. We used a different color scale between the two preclinical models to discriminate and show the dose distributions differences. Highly conformal dose coverage of the bone marrow sites was achieved in SOC-TMI preclinical model, as shown in **Figure 3B**. The dose distribution to the target and OARs (lungs and liver) in the transverse image at the level of the mediastinum in both SOC-preclinical and 3D-preclinical models is shown in **Figures 3C, D**. Moreover, SOC-TMI compared with 3D-TMI has further reduced the body integral dose by an average of 17.6% ± 6.2%.

**Figure 4:**
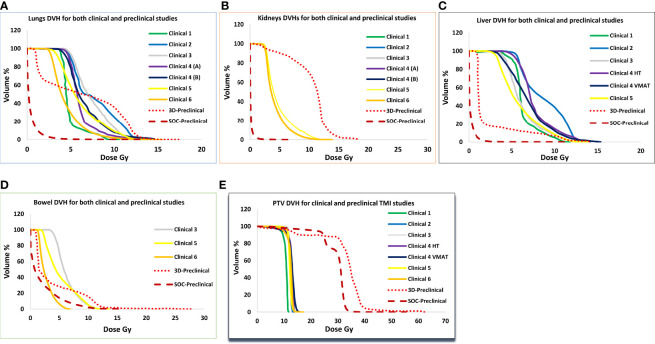
Comparison of average dose volume histograms (DVH) for the **(A)** lungs, **(B)** kidneys, **(C)** liver, **(D)** bowel, and **(E)** PTV between six clinical TMI plans, 3D-preclinical model (red dotted lines), and the SOC-preclinical TMI model (dark red dashed lines).

**Figure 4** shows dose volume histograms for PTV and various organs from six clinical TMI studies, 3D-preclinical TMI, and SOC-preclinical TMI treatments (**Figures 4A–E** **)**. DVH indicated the successful sparing of the major normal organs of the SOC-preclinical TMI. The SOC-preclinical TMI (dark red dashed lines) showed a significant reduction in dose exposures for various OARs compared to 3D-preclinical TMI and clinical studies’ dose levels. PTV is covered by 85%–95% of the prescribed dose in all six different clinical studies. Preclinical PTV covered by 85% of the prescribed dose in both 3D-TMI and SOC-TMI models.

## Discussion

The TMI treatment technique is increasingly becoming an alternative to TBI for conditioning regimens for hematopoietic cell transplantation because it reduces radiation exposure to all organs. Dose-escalated TMI has been successfully implemented with improved survival (24). Subsequently, TMI preclinical models (2D and 3D) were developed to enhance our understanding of the role of TMI in hematological malignancies (15, 16). Through a detailed comparative evaluation of dosimetric coverage, we observed that lungs and kidney proximal to the skeletal target received relatively higher doses in preclinical TMI than in clinical TMI. One of the most important late effects of higher doses to the OAR is lung pneumonitis (25–30). Lung pneumonitis is known to be the major dose-limiting factor and has been reported to correlate with the mean lung dose (25–28). Thus, sparing normal tissues while maintaining dose conformality to the target might further reduce the normal tissue complications, and thus, there is a need for evaluating detail of OAR dosimetry and adaptation of technology to reduce/vary dose to organs/tissues. IMRT is widely used for conformal dose delivery in the clinic and is being adopted for TMI. However, performing IMRT for small animal experiments to closely mimic human clinical scenarios faced insurmountable engineering challenges. To mitigate the discrepancy and make the preclinical model more translatable, we evaluated the next-generation preclinical TMI using SOC for enhanced dosimetric conformality. SOC system is designed and fabricated to be the first general-purpose small animal IMRT platform.

To this end, we compared the dosimetric results of clinical TMI studies, 3D-TMI preclinical model, and SOC-TMI preclinical model. PTV of both clinical and preclinical TMI studies showed good coverage. DVH of PTV in preclinical TMI models showed a relatively high dose to bone medium due to the photoelectric absorption of low energy x-ray (effective energy of 78.8 keV) (17). The 3D-TMI showed larger dose heterogeneity in comparison to SOC-TMI. This is because there is limited available collimator to accurately cover the irregular shaped geometry that causes overlap in some regions, leading to some hot spots and less coverage in some regions, leading to the cold spot. On the other hand, SOC-TMI uses automated and varied rectangular apertures to cover the target, and reducing the heterogeneity.

Clinical TMI studies showed a large dose variation in organs between centers. This is potentially due to the difference in dose constraints, treatment machine, treatment techniques, patient positioning, etc. Although the 3D-TMI preclinical model was shown to be equivalent to the clinical TMI (16), the dose to the lungs and kidneys was relatively higher due to their proximity to the spine and lower 3D plan dose conformity. The SOC-TMI method is based on RAO planning (31) and SOC dose modulator (22, 32) for IMRT planning. Compared with the MLC-based IMRT, SOC uses substantially fewer leaves while maintaining the modulation resolution. Therefore, SOC is more conducive to miniaturization. The performance of SOC for preclinical small-field radiation has been physically demonstrated. Moreover, SOC-TMI preclinical model showed a low integral dose to the body compared with the 3D-TMI preclinical model. Whether SOC-TMI delivery can improve integral dose may also be an important question, particularly in association with secondary cancer (33). As reported by D’Souza (34) in solid tumors, beam margin size and beam energy are the most relevant parameters, with smaller margins and higher energy consistently reducing the integral dose to the body regardless of the number of beams. In the two preclinical models, given the same beam energy of 225 keV (effective energy of 78.8-keV x-ray), one would expect that the smaller margin used in SOC-TMI model because of the shaped rectangular collimators leads to a reduction of the integral dose. The 3D-TMI model dose delivery scheme increases the (normalized) average dose to the body, thus increasing the integral dose as well. According to the two TMI model planning, number of beams, beam direction, and relative beam weight have little effect on the integral dose. The superior SOC-TMI dosimetry is evident in this study.

Compared with the 3D-TMI technique utilizing parallel-opposed beams with manually created conformal fields, SOC-TMI presents several major technological advances. The SOC-TMI model will help to automatize treatment planning and delivery and to achieve a more conformal and homogeneous target dose. The SOC-TMI will also allow varying radiation doses for each organ at risk for a larger range than the current clinical and preclinical system. It will help us to enhance scientific knowledge, namely, i) obtaining a radiobiological correlation of dose versus tissue damage and their impact on the tissue repair process and ii) understanding how varying radiation exposure to organs could impact engraftment. Our recent study suggests that a very low body dose may adversely impact engraftment (16). However, little is known on how dose variation stimulates factors (inflammatory cytokines, growth factors, etc.) that support engraftment. This knowledge is essential to developing an optimal radiation conditioning to achieve stable engraftment as well as reduced toxicity. iii) There is clinical concern that reduced doses to organs may reduce treatment efficacy because of the systemic nature of the hematological disease. Therefore, our initial clinical TMI development was to maintain a certain low level of organ dose (to prevent increased toxicity) while increasing BM-specific radiation to enhance the antileukemic effect. Therefore, clinical question of preferred dose to specific organs is not settled. This will require further investigation using preclinical TMI in BMT and disease models. Such knowledge radiation and biology will strengthen developing a rationale for translation in future clinical trials which may require further improvement in conformal radiation delivery by available clinical machines.

The data presented in this study demonstrate the versatility of SOC technology in providing exceptional target coverage and OAR sparing capabilities for difficult techniques like TMI. SOC-TMI preclinical model allowed a high-precision dose optimization for targets such as bone, bone marrow, and spleen and non-target vital organs. SOC-TMI preclinical has statistically improved the TMI plan quality. In addition, the SOC-TMI plans could deliver TMI treatment in an efficient manner in terms of treatment time. The overall SOC-TMI treatment planning time was approximately 45 min, which is 40% lower than the treatment time of the 3D-TMI model (~75 min). This offers a flexibility to tailor the treatment delivery within a reasonable amount of time. This novel RAO for SOC preclinical planning will substantially advance the preclinical radiation research and reduces the gap in treatment plan quality between clinical and preclinical radiotherapy, potentially increasing the translatability of small animal studies.

Despite the advantages of SOC-TMI, the current SOC-TMI has several limitations. i) Tissue heterogeneity is not incorporated in the model. Corrections were made for bone and lungs based on their density table. ii) Availability of SOC hardware is limited, preventing wide adoption and actual testing of SOC-TMI. However, this simulation shows dosimetric advantages for installing and commissioning of SOC-TMI in the future.

## Conclusion

The preliminary results of SOC-TMI preclinical model are promising. SOC-TMI dosimetry result shows that this technique offers many attractive advantages. SOC-TMI preclinical model could be used as a new method for delivering TMI with high accuracy. SOC-TMI preclinical model demonstrates excellent target dose conformity and the ability to avoid unnecessary doses to critical structures adjacent to the target volumes. In addition to the lungs and kidneys, substantial radiation dose reductions to all sensitive structures are possible with this new technique of a SOC for small animal intensity-modulated TMI therapy.

## Data Availability Statement

The original contributions presented in the study are included in the article/supplementary material. Further inquiries can be directed to the corresponding author.

## Author Contributions

AA designed the study, collected, analyzed data, and wrote the paper. LJ and KS calculated dose for SOC-TMI and wrote the manuscript. DZ, AL, SM, HG, JW, SS, CF, CZ, and AP collected data and aided in the results. CA edited the manuscript. SH designed, wrote the manuscript, provided guidance. All authors contributed to the article and approved the submitted version.

## Funding

Research reported in this publication is supported by the National Institutes of Health (2R01CA154491-01 to SH and R01CA259008 to KS).

## Conflict of Interest

The authors declare that the research was conducted in the absence of any commercial or financial relationships that could be construed as a potential conflict of interest.

## Publisher’s Note

All claims expressed in this article are solely those of the authors and do not necessarily represent those of their affiliated organizations, or those of the publisher, the editors and the reviewers. Any product that may be evaluated in this article, or claim that may be made by its manufacturer, is not guaranteed or endorsed by the publisher.
